# Il muscolo come organo endocrino: focus su irisina

**DOI:** 10.1007/s40619-022-01177-3

**Published:** 2022-11-11

**Authors:** Carla Giordano, Roberta Amodei, Claudia Di Stefano

**Affiliations:** 1 Insegnamento di Endocrinologia, Dipartimento Promozione della Salute, Materno-Infantile, di Medicina Interna e Specialistica di Eccellenza “G. D’Alessandro”, Università degli Studi di Palermo, Palermo, Italia; 2 U.O.C. Malattie Endocrine, del Ricambio e della Nutrizione, A.U.O.P. “P. Giaccone”, Palermo, Italia

**Keywords:** Irisina, Termogenesi, Irisina e muscolo, Irisina e metabolismo

## Abstract

Il muscolo scheletrico agisce come organo endocrino che, in risposta all’esercizio, produce e secerne centinaia di miochine che agiscono in maniera autocrina, paracrina ed endocrina con effetti sul metabolismo lipidico e glucidico, formazione ossea, funzione endoteliale e crescita tumorale. Negli ultimi anni, numerosi studi hanno concentrato la loro attenzione su una nuova proteina, l’irisina, adipomiochina in grado di indurre cambiamenti nel tessuto adiposo, attivare la termogenesi e fungere da collegamento tra muscoli e altri tessuti del corpo.

**Informazioni Supplementari:**

La versione online contiene materiale supplementare disponibile su 10.1007/s40619-022-01177-3.

## Introduzione

Il muscolo scheletrico è l’organo più esteso del corpo umano ed è composto da fibre contrattili costituite da miociti. In aggiunta alle più note capacità di stabilità e movimento, in risposta all’esercizio fisico i miociti sono in grado di secernere alcune sostanze come chemochine, citochine e miochine, in grado di regolare diversi processi metabolici in vari tessuti e organi, quali fegato, ossa, cervello e tessuto adiposo, attraverso vie di segnalazione endocrine, paracrine o autocrine. Alla luce di quanto detto, il muscolo scheletrico può essere classificato come un vero e proprio organo endocrino.

Le miochine dagli effetti biologici più noti sono l’interleuchina 6 (IL-6), la proteina chemiotattica monocitaria 1 (MCP1), il fattore di crescita simile all’insulina-1 (IGF-1) e la miostatina.

Sebbene la funzione biologica sia stata descritta solo per il 5% di tutte le miochine conosciute, la loro identificazione ha portato a ipotizzare che alcuni effetti benefici dell’esercizio sulle malattie metaboliche possano essere correlati alle miochine e alle loro interazioni con altri sistemi. Pertanto, le miochine possono essere utili biomarcatori nel monitoraggio di alcune condizioni patologiche come il cancro, il diabete e le malattie neurodegenerative.

Nel 2012, Boström e collaboratori riportavano la scoperta di una nuova molecola – secreta dai miociti di topi transgenici che sovraesprimevano il gene *Ppargc1a* codificante il PPAR$\gamma $ coattivatore-1$\alpha $ (PGC1$\alpha $) – in grado di indurre cambiamenti nel tessuto adiposo, attivare la termogenesi [1] e fungere da mezzo di comunicazione tra i muscoli e altri tessuti. In considerazione delle sue note capacità di *cross talk* tra muscolo e tessuto adiposo, questa molecola è stata chiamata irisina, in riferimento alla dea greca Iris, messaggera degli dei [2].

Dalla sua scoperta, l’irisina è stata oggetto di numerosi studi che hanno consentito di approfondire le sue proprietà pleiotropiche.

**Figure 1:**
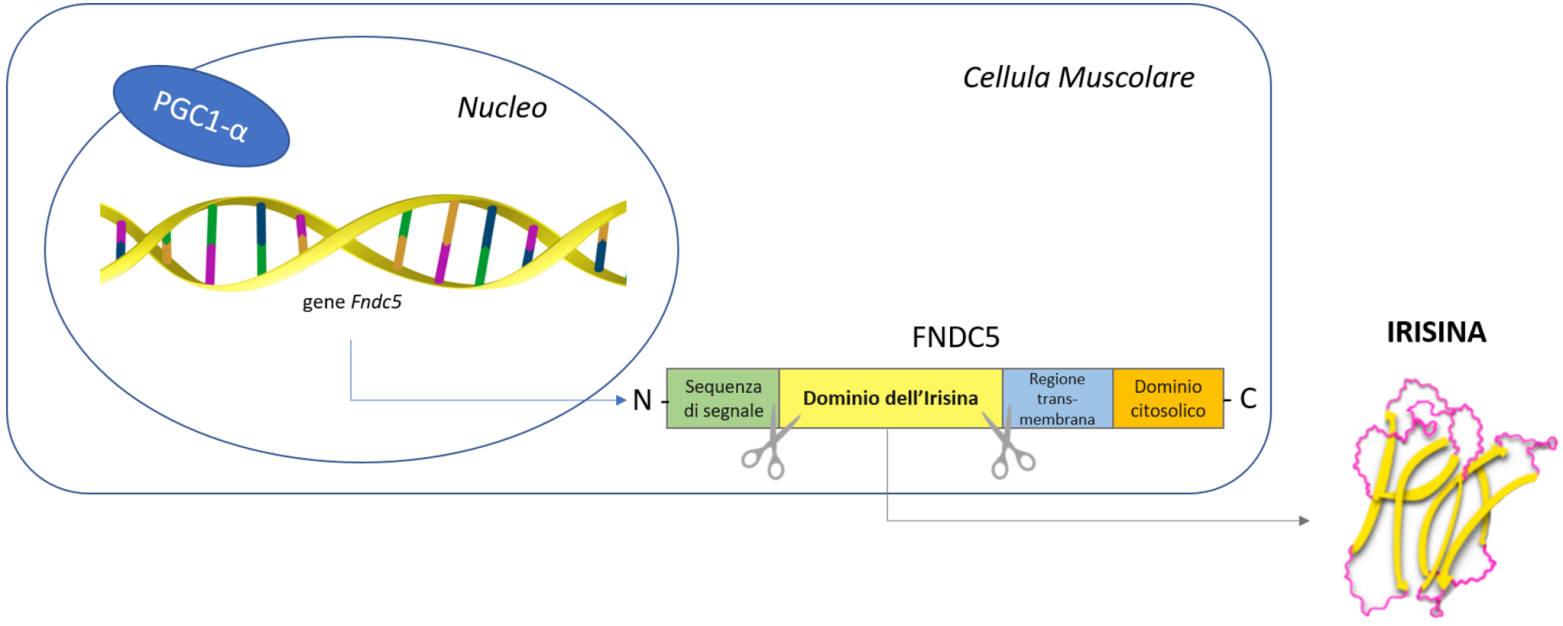

L’attività contrattile è l’elemento regolatore chiave per l’espressione e la secrezione delle miochine, compresa l’irisina. La contrazione della fibra muscolare durante e dopo l’esercizio fisico induce l’attivazione di PGC1-$\alpha $, che regola l’espressione del gene *Fndc5*, il cui prodotto è appunto la proteina FNDC5 [3]. Dopo la scissione proteolitica, la glicosilazione e la dimerizzazione di FNDC5, viene rilasciata una nuova proteina, l’irisina (Fig. 1), costituita da una sequenza di 112 aminoacidi, identica nell’uomo e nel topo [1].

Nell’uomo, *Fndc5* è altamente espresso nel muscolo scheletrico. L’espressione genica di FNDC5 e, quindi, la correlazione con la produzione di irisina, in altri tessuti necessita tuttavia di ulteriori indagini. L’mRNA di FNDC5 sembra essere ampiamente espresso in varie aree cerebrali e muscolari (sia cardiache che scheletriche). Tessuto adiposo bruno (BAT), prostata, intestino, pancreas e fegato (anche nell’uomo) mostrano livelli di espressione moderati. Altri tessuti, come tessuto adiposo bianco, polmone, rene, timo, milza, placenta, stomaco e fegato (nei ratti), hanno livelli di espressione molto bassi o non rilevabili. Ci sono sia analogie che evidenti differenze nell’espressione genica di FNDC5, tra i tessuti animali e tessuti umani, per questo sono necessari ulteriori studi in merito [4].

In considerazione di quanto detto, i dati relativi ai livelli di irisina nel plasma e nel siero non possono essere attualmente utilizzati per la valutazione di una gamma indicativa di valori considerati “normali”. Inoltre, l’irisina può presentarsi sia in forma glicosilata che non glicosilata, generando ulteriore confusione nell’interpretazione dei suoi livelli ematici. Non è ancora chiaro in quale forma esplichi la sua funzione e se questo moduli il suo effetto in diverse condizioni fisiopatologiche. Alcuni studi, infatti, hanno indicato che la glicosilazione dell’irisina influenzi la sua efficacia.

**Figure 2:**
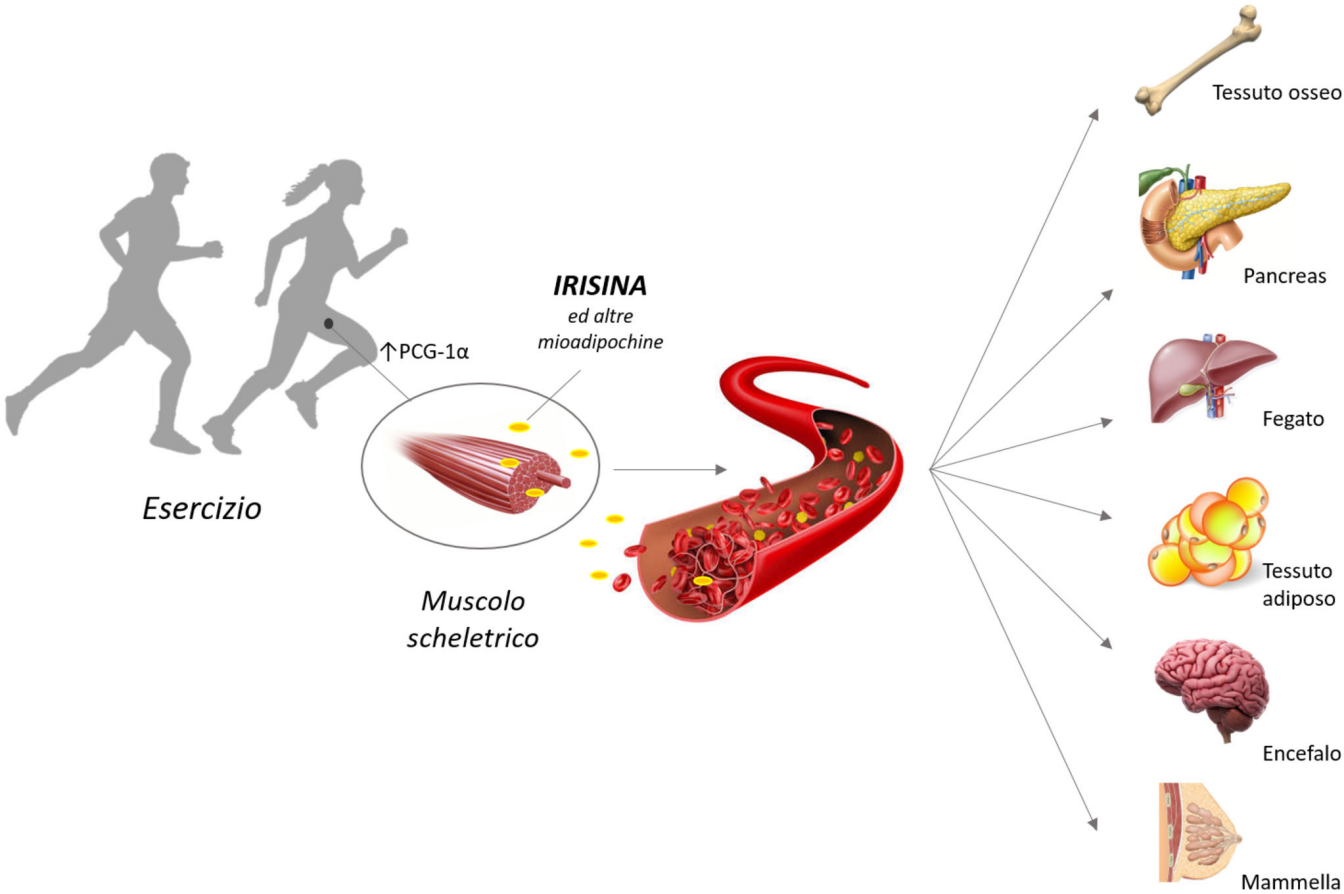

L’irisina svolge diverse azioni su diversi organi e tessuti e numerose sono le funzioni del nostro organismo in cui essa interviene, assumendo un ruolo importante nell’obesità, nelle malattie metaboliche e ossee, nel sistema nervoso centrale (SNC) e nel cancro (Fig. 2).

Tra le sue funzioni più note c’è quella di stimolare il *browning* del tessuto adiposo bianco (WAT) e la termogenesi [5]. Dopo la scoperta dell’irisina, infatti, è stato dimostrato che l’iniezione di un anticorpo anti-FNDC5 nei topi 10 giorni prima del test del nuoto, ha ridotto l’espressione della proteina disaccoppiante 1 o termogenina (UCP1). Questo ha suggerito, per la prima volta, che il coinvolgimento del *pathway* dell’irisina sia necessario a spiegare, almeno in parte, l’effetto dell’esercizio nell’innescare il meccanismo di browning [1]. Esperimenti condotti nei topi e poi nell’uomo hanno dimostrato che l’esercizio fisico è correlato a un aumento dell’mRNA di PGC-1$\alpha $ e FNDC5 e, conseguentemente, ai livelli di irisina circolanti [1].

Contrastanti sono invece i dati relativi ai livelli di irisina nel diabete e negli stati di obesità: molti studi hanno riportato livelli di irisina circolanti bassi in questi stati patologici ma le evidenze più recenti suggeriscono come nell’obesità si osservi una condizione di resistenza all’irisina e livelli di quest’ultima conseguentemente più elevati. In ogni caso, è stato dimostrato come in queste condizioni patologiche l’attività benefica complessiva dell’esercizio fisico possa agire (almeno in parte) modulando i livelli di irisina e ripristinando i valori fisiologici [5]. Studi sull’uomo sembrano indicare una diversa efficacia nella stimolazione della via di irisina, in relazione al tipo di esercizio fisico. Il maggiore aumento dei livelli di irisina durante l’esercizio sub-massimale rispetto allo sforzo fisico massimo ha suggerito che l’esercizio di resistenza può essere uno stimolo più potente per la secrezione di irisina [5].

Tra le altre funzioni riconosciute dell’irisina è riportata la capacità di apportare benefici sulla funzione cognitiva promuovendo la neuroprotezione contro i danni da accumulo di $\beta $-amiloide (A$\beta $). È stato dimostrato, inoltre, che l’irisina ricombinante (R-irisina) abbia effetti benefici nell’osteoporosi e sui cardiomiociti dopo infarto del miocardico (MI) [6].

Sembrerebbe altresì che l’espressione di FNDC5/irisina sia regolata da peptidi come l’insulina, il glucagone e la leptina e come l’irisina, a sua volta, potrebbe influenzare l’omeostasi del glucosio. Tuttavia, molti aspetti relativi alla regolazione dell’espressione genica di FNDC5 e la relativa produzione di irisina, sia nel SNC che nei tessuti periferici, non sono ancora ben compresi [4].

## Irisina, obesità e termogenesi

Il primo ruolo riconosciuto all’irisina riguarda la regolazione del metabolismo lipidico. L’irisina permette, infatti, di ridurre la quota di grasso corporeo inducendo il *browning*, ovvero la conversione del tessuto adiposo bianco (WAT) (dove vengono immagazzinati i trigliceridi) in tessuto adiposo bruno (BAT). Attraverso l’espressione di UCP-1, aumenta la densità cellulare dei mitocondri [7], favorendo il dispendio energetico, il calo ponderale e riducendo, quindi, l’insulino-resistenza legata all’obesità, con conseguente miglioramento dell’omeostasi glucidica.

Il WAT è considerato la seconda fonte più importante di irisina dopo il muscolo scheletrico. La FNDC5/irisina derivata da WAT può rappresentare fino al 30% circa dei suoi livelli circolanti e, analogamente al muscolo scheletrico, la sua secrezione è aumentata dopo esercizio fisico.

Il bersaglio principale dell’irisina è rappresentato dal tessuto adiposo, con effetti differenti in relazione alla specie (uomo o roditori) e al tipo di adipocita.

Nell’uomo, ad esempio, la riduzione delle concentrazioni di irisina secondaria a un maggiore apporto energetico è coerente con gli effetti metabolici dannosi dell’eccesso di cibo.

Gli effetti sul *browning* dell’irisina nell’uomo sono meno consolidati rispetto ai roditori. Negli adipociti umani maturi, l’irisina stimola l’imbrunimento, indicato da un aumento di UCP1 e PR/SET Domain 16 (PRDM16), a differenza di quanto accade nei preadipociti maturi in cui, al contrario, diminuisce l’espressione dei geni collegati al browning. Inoltre, l’irisina stimola la glicolisi [8].

Diversi sono gli studi che hanno cercato di dimostrare sull’uomo le potenziali correlazioni tra i livelli di irisina circolanti e l’obesità. La maggior parte di questi hanno riportato livelli di irisina circolanti positivamente associati all’indice di massa corporea (BMI), al peso, alla massa grassa, alla circonferenza vita, al rapporto vita-fianchi e alla massa muscolare [9].

La correlazione tra livelli di irisina aumentati e obesità può essere spiegata come probabile meccanismo controregolatorio volto a favorire un maggior dispendio energetico e a migliorare la sensibilità all’insulina. Potrebbe, inoltre, essere spiegata da un verosimile meccanismo di resistenza all’azione dell’irisina, simile all’insulina e alla leptina-resistenza osservate nell’obesità e nel diabete mellito di tipo 2 [10].

## Irisina e attività fisica

Oltre a questi effetti, l’irisina regola positivamente anche il metabolismo muscolare. I miociti trattati con irisina *in vitro* esprimono livelli più elevati di PGC-1$\alpha $, fattore respiratorio nucleare 1 (NRF-1), fattore di trascrizione mitocondriale A (TFAM), trasportatore del glucosio 4 (GLUT4), UCP3 e irisina, il che implica un circuito di autoregolazione positivo tra PGC-1$\alpha $ e FNDC5 [11]. In tal modo, il dispendio energetico e il metabolismo ossidativo nelle cellule muscolari sono positivamente regolati dall’irisina. L’irisinemia aumenta in seguito a contrazione muscolare ma, come per altri fattori, la regolazione dell’irisina potrebbe dipendere dal protocollo di allenamento specifico, da intensità, tempo del prelievo dopo l’esercizio, età, sesso, etnia, forma fisica e altre variabili.

I dati in letteratura confermano che i diversi tipi di attività fisica possono aumentare la produzione di irisina, sebbene il picco maggiore sia dopo esercizio di tipo aerobico [3] e in funzione dell’intensità [9], anche se solo a breve termine, calando dopo circa 30 minuti dalla fine dell’esercizio. Inoltre, la secrezione dell’irisina sembra avere un particolare ritmo circadiano, con livelli minori al mattino e un picco intorno alle ore 21:00.

Da uno studio del 2018 è emerso che le concentrazioni di irisina aumentino gradualmente con il livello di attività fisica abituale (HPA) [12].

## Irisina e metabolismo osseo

Sembra che l’irisina sia coinvolta nell’aumento della densità minerale ossea (BMD). L’attività fisica permette, appunto, un aumento della BMD e regola l’equilibrio tra formazione e riassorbimento osseo, promuovendo la differenziazione osteoblastica, prevenendo la perdita ossea, riducendo il numero di osteoclasti e migliorando, dunque, struttura e resistenza ossea [13]. Concentrazioni di irisina ridotte, appunto, sono state riscontrate in donne in età post-menopausale che avevano subito fratture osteoporotiche.

Queste scoperte potrebbero aprire nuove frontiere per il trattamento dell’osteoporosi.

## Irisina e neurogenesi

È stato dimostrato che il cervello umano esprime l’irisina [14]. Aumentando i livelli di irisina circolanti, l’esercizio di resistenza induce l’espressione di FNDC con conseguente incremento del fattore neurotrofico *Brain-derived neurotrophic factor* (BDNF) che, a sua volta, regola la differenziazione e la sopravvivenza neuronale, promuovendo lo sviluppo di nuove cellule nervose, l’incremento del numero di sinapsi e potenziando funzioni cognitive come memoria e apprendimento [15].

In particolare, l’irisina e il suo precursore FNDC5 sono espresse dalle cellule di Purkinje del cervelletto e nel nucleo paraventricolare, dove viene espressa una proteina implicata nella regolazione del senso della fame, il neuropeptide Y. Quindi, il ruolo dell’irisina sul metabolismo potrebbe essere determinato anche da questa sua azione a livello centrale.

Una possibile implicazione terapeutica dell’irisina potrebbe riguardare le patologie neurodegenerative caratterizzate da declino cognitivo, prima tra tutte la malattia di Alzheimer, ma anche patologie correlate a riduzione della neurogenesi, come schizofrenia e depressione maggiore.

## Irisina e metabolismo

Da studi su animali di laboratorio, l’irisina migliora la tolleranza al glucosio e riduce l’insulinemia a digiuno [1]. Inoltre, la concentrazione di irisina è ridotta in diabetici di tipo 2, se confrontati con pazienti con normale tolleranza glucidica, e i valori sono ulteriormente ridotti in caso di complicanze, sia macro che microvascolari [7–16], suggerendo un possibile ruolo dell’irisina come marker di malattia macrovascolare nel diabetico. Di contro, in altri studi i livelli sierici di irisina sono stati descritti essere più elevati in soggetti con DM2 [17] e avere una relazione inversa con la sensibilità insulinica [18], interpretabile come tentativo dell’irisina di contrastare l’insulino-resistenza, oppure come possibile resistenza all’irisina. Un polimorfismo del gene FNDC5, infine, sembra proteggere dal DM2 [19].

L’irisina potrebbe proteggere dalla steatosi epatica non alcolica (NAFLD), prevenendo l’accumulo lipidico all’interno degli epatociti, attraverso la regolazione della lipogenesi [20]. Tuttavia, è stata riscontrata una correlazione positiva tra irisina sierica e NAFLD, anche direttamente proporzionale alla gravità della NAFLD [21].

Molti studi hanno evidenziato una relazione inversa tra irisina e indice di massa corporea (BMI) [22, 23], mentre altri hanno rilevato livelli di irisina correlati con peso, BMI, circonferenza vita e massa grassa misurata mediante bioimpedenziometria [9], evidenziando una discesa dell’irisinemia dopo calo ponderale e rialzo in caso di nuovo aumento.

Si è visto, inoltre, che pazienti affetti da sindrome metabolica presentavano livelli di irisina minori rispetto a controlli sani [24]. Da ciò si è dedotto un possibile ruolo protettivo dell’irisina nei confronti dell’insulino-resistenza e della sindrome metabolica in generale.

## Irisina e rischio cardiovascolare

È stata rilevata un’iperirisinemia in soggetti con pressione arteriosa, prevalentemente diastolica, più elevata [16–18]. Inoltre, valutando il rischio globale secondo i criteri di Framingham, livelli maggiori di irisina erano associati a un rischio aumentato a 10 anni [18]. Diversi autori, invece, hanno ipotizzato un ruolo favorevole dell’irisina sulla funzione endoteliale, un predittore indipendente di eventi cardiovascolari [23, 25, 26], collegando concentrazioni ematiche ridotte con disfunzione e danno endoteliale, aumento dello spessore mio-intimale carotideo e maggiore tendenza all’aterosclerosi [27]. Rispetto all’omocisteinemia, il cui aumento rappresenta un fattore di rischio cardiovascolare, l’irisina sembra pertanto mantenere un rapporto inversamente proporzionale. In seguito a studi condotti in soggetti con infarto miocardico acuto (IMA), si è prospettata la possibilità di utilizzare l’irisina come marker precoce di IMA, in quanto sono stati riscontrati bassi livelli in circolo dopo IMA, ma ciò deve essere ulteriormente approfondito [28].

## Irisina e cancro

I risultati più interessanti riguardano il carcinoma della mammella, le cui cellule vengono indotte all’apoptosi da parte dell’irisina riducendo, inoltre, la vitalità e la capacità diffusiva delle cellule neoplastiche e aumentando la risposta alla chemioterapia [29]. Inoltre, è stato suggerito un impiego come biomarcatore, in quanto le donne affette presentano concentrazioni di irisina ridotte [30].

Allo stesso modo, pazienti affetti da carcinoma del colon-retto presentano concentrazioni inferiori di irisina mentre livelli ematici maggiori sembrano ridurre il rischio di neoplasia, suggerendo un ruolo protettivo [31].

Di contro, studi su carcinomi epatocellulari hanno ipotizzato come la molecola possa indurre proliferazione e invasività tumorale [32], suggerendo l’ipotesi che l’irisina possa avere ruoli diversi in tumori diversi.

## Irisina e diabete mellito tipo 1

Esiste una correlazione positiva tra glicemia e irisina. L’iperirisinemia nel diabete mellito tipo 1 (DM1) sembra essere, infatti, una risposta compensatoria all’iperglicemia [33, 34], probabilmente poiché l’iperglicemia causa la produzione di ROS e questi, a loro volta, potrebbero stimolare la produzione di irisina al fine di ridurre lo stress ossidativo.

Inoltre, è stato dimostrato come l’irisina stimoli negli adipociti la produzione di betatrofina, un ormone in grado di indurre la proliferazione della $\beta $-cellula pancreatica [7, 35].

Infine, è stato dimostrato come uno scarso controllo glicemico possa influenzare la struttura dell’osso e che nel DM1 è riscontrabile una riduzione della BMD. Valutando la relazione tra DM1 e aumentato rischio di frattura, esiste una correlazione negativa tra irisina, HbA1c e vitamina D, e una positiva con la BMD e con l’osteocalcina, un marker di rimodellamento osseo [36]. Nei pazienti diabetici con un migliore controllo metabolico e del turnover osseo, gli elevati livelli di irisina predicono un migliore controllo glicemico e una maggiore salute ossea, aprendo prospettive future verso l’impiego di irisina esogena in questi pazienti o, comunque, verso l’incremento dell’esercizio fisico allo scopo di elevarne le concentrazioni in circolo. L’irisina potrebbe rappresentare, inoltre, in futuro un indicatore prognostico, permettendo di valutare la gravità del quadro e la risposta al trattamento.

## Irisina e malattie endocrine

L’irisinemia è stata riportata come ridotta in pazienti con tiroidite di Hashimoto in fase ipotiroidea, rispetto agli eutiroidei, correlata positivamente con l’fT4 e negativamente con TSH e CPK sierica, dal momento che il sistema muscolare scheletrico è un importante bersaglio degli ormoni tiroidei ed è anche coinvolto nella produzione di irisina [37]. L’abbassamento dei livelli di irisina potrebbe essere dovuto al venir meno dell’azione di stimolo di fT3 e fT4 sul PGC-1$\alpha $ [38]. Dopo terapia e raggiungimento dell’eutiroidismo i livelli di irisina sono apparsi aumentati e quelli di CPK ridotti [38, 39].

Confrontando pazienti con ipotiroidismo di lunga durata (AITD), di breve durata (TC) e controlli sani, i livelli di irisina sono risultati minori nel gruppo AITD sia rispetto ai TC che ai sani, in assenza di differenze nei livelli di fT3 e fT4 tra i due gruppi di pazienti ipotiroidei. Al contrario, la CPK era maggiore negli ipotiroidei rispetto ai sani e inversamente proporzionale ai livelli di irisina. In patologie di nuova insorgenza, un danno muscolare libererà più alte quantità di irisina; a lungo termine il danno muscolare sarà così avanzato da ridurre le capacità di produzione di irisina [40].

L’obesità, l’irisinemia e i valori di fT4 sembrano essere fattori di rischio indipendenti per l’ipotiroidismo [41], mentre il TSH si è mostrato essere un predittore indipendente dei livelli di irisina [42].

Da uno studio del 2019 effettuato su bambini con deficit di GH, la terapia sostitutiva ha portato a un aumento dei livelli di irisina, a sua volta fortemente correlato a una diminuzione di circonferenza vita e BMI e aumento dei livelli di IGF-1, confermando un effetto positivo della terapia sostitutiva con rGH [43].

Recenti evidenze dimostrano come nei pazienti con Sindrome di Klinefelter i livelli di irisina siano più elevati rispetto ai controlli sani, e questi sono direttamente correlati con il grado di insulinoresistenza [44].

## Irisina e COVID-19

La malattia da coronavirus, dichiarata pandemia dall’OMS a marzo 2020, ha indirizzato la comunità scientifica a esaminare con crescente attenzione le correlazioni esistenti tra l’infezione da COVID-19 e i diversi aspetti fisiopatologici che, a sua volta, possono influenzarne il decorso.

È stato ipotizzato che l’irisina abbia proprietà antinfiammatorie in virtù delle sue provate capacità di ridurre la produzione di specie reattive dell’ossigeno (ROS) e modulare l’attività macrofagica in senso antinfiammatorio [45].

Di contro, l’obesità, condizione che rende i pazienti più suscettibili a infezioni gravi da SARS-Cov-2, è associata a un pattern citochinico pro-infiammatorio.

Da uno studio pubblicato da De Oliveira e collaboratori nel 2020, sembrerebbe che l’irisina presenti, su colture di adipociti umani, un effetto positivo nella regolazione di diversi geni correlati all’esito più o meno favorevole di COVID-19. Nello specifico, è stato evidenziato come il trattamento con irisina causi la riduzione dell’espressione di alcuni geni implicati nella replicazione virale (FURIN, ADAM10, TLR3, KDM5B e SIRT1) e, di contro, aumenti l’espressione di geni che la bloccano (TRIB3) con presumibile effetto di riduzione del tasso di infezione da SARS-CoV-2 e ipotizzabili effetti positivi sull’andamento della malattia, se valutati in aggiunta agli effetti antinfiammatori dell’irisina [46].

Questi promettenti risultati se potenziati da ulteriori evidenze, potrebbero rappresentare uno punto di partenza per lo sviluppo di strategie terapeutiche utili (Tabella 1).

**Table 1:** 

**Tessuto adiposo**	- *Browning* del tessuto adiposo bianco in tessuto adiposo bruno
	- Aumento della termogenesi mediante modulazione dell’espressione della proteina disaccoppiante -1 (UCP)
**Tessuto osseo**	- Promozione della differenziazione in senso osteoblastico
	- Miglioramento della BMD, struttura e resistenza ossea
**Tessuto nervoso**	Attraverso la modulazione dell’espressione di BDNF:
	- differenziazione e sopravvivenza neuronale
	- aumento del numero di sinapsi
	- potenziamento di alcune funzioni cognitive come memoria e apprendimento
**Cancerogenesi**	Carcinoma della mammella
	- promozione dell’apoptosi
	- riduzione della vitalità delle cellule tumorali
	- riduzione della capacità diffusiva
	- aumentata risposta alla chemioterapia
	Carcinoma epatocellulare
	- promozione della proliferazione cellulare
	- promozione dell’invasività tumorale
**Diabete mellito di tipo 1**	- Attraverso la produzione di betatrofina da parte degli adipociti, promozione della proliferazione beta-cellulare pancreatica
	- Livelli maggiori di irisina correlano con miglior controllo glicemico
**Funzione tiroidea**	Probabile fattore di rischio indipendente (insieme all’obesità e ai livelli di FT4) per lo sviluppo di ipotiroidismo
**Rischio cardiovascolare**	Probabile effetto favorevole sulla funzione endoteliale

## Conclusioni

Come già esposto, le miochine forniscono una spiegazione molecolare dell’ampio *cross-talk* esistente tra muscolo e altri tessuti, consacrando di fatto il muscolo a vero e proprio organo endocrino. Molteplici e talvolta discordanti sono in atto le funzioni attribuite all’irisina; pertanto, lo studio delle miochine è meritevole di ulteriori approfondimenti negli anni a venire, al fine di identificare strategie terapeutiche utili in diversi ambiti clinici.

## Informazioni Supplementari

A seguire il link al materiale elettronico supplementare.

## Dichiarazioni etiche

### Conflitto di interesse

Le autrici Carla Giordano, Roberta Amodei e Claudia Di Stefano dichiarano di non avere conflitti di interesse.

### Consenso informato

Lo studio presentato in questo articolo non ha richiesto sperimentazione umana.

### Studi sugli animali

Le autrici di questo articolo non hanno eseguito studi sugli animali.
