# Supplementary material for: Il muscolo come organo endocrino: focus su irisina
Source: L'Endocrinologo. 2022 Nov 11;23(6):579–86. [Article in Italian] doi: 10.1007/s40619-022-01177-3 (PMC9650659; doi:10.1007/s40619-022-01177-3)
Supplement: Supplementary file 1 [file 40619_2022_1177_MOESM1_ESM.doc]

**Scheda di autovalutazione**

**1. Quando è stata scoperta l’irisina?**

a. nel 2002

b. nel 2008

c. nel 2012

**2. Qual è la proteina precursore dell’irisina?**

a. FNDC5

b. PGC-1α

c. PPAR-γ

**3. Quale tra queste è una delle funzioni più note dell’irisina?**

a. ridurre la termogenesi

b. favorire il *browning* del tessuto adiposo bianco

c. promuovere la sintesi dei ROS

**4. Sul tessuto osseo l’irisina promuove:**

a. il differenziamento in senso osteoclastico

b. il differenziamento in senso osteoblastico

c. non sono noti gli effetti dell’irisina sull’osso
